# 25-Hydroxycholesterol is involved in the pathogenesis of amyotrophic lateral sclerosis

**DOI:** 10.18632/oncotarget.14416

**Published:** 2017-01-02

**Authors:** Sung-Min Kim, Min-Young Noh, Heejaung Kim, So-Young Cheon, Kang Mi Lee, Jaeick Lee, Eunju Cha, Kyung Seok Park, Kwang-Woo Lee, Jung-Joon Sung, Seung Hyun Kim

**Affiliations:** ^1^ Department of Neurology, Seoul National University, College of Medicine, Seoul, Korea; ^2^ Department of Neurology, Seoul National University Bundang hospital, Seong Nam, Korea; ^3^ Department of Neurology, Hanyang University, College of Medicine, Seoul, Korea; ^4^ Doping Control Center, Korea Institute of Science and Technology, Korea

**Keywords:** amyotrophic lateral sclerosis, cholesterol, hydroxycholesterol, 25-hydroxycholesterol, liver X receptor

## Abstract

This study aimed to evaluate the levels of three major hydroxycholesterols (24-, 25-, and 27-hydroxycholesterols) in the serum and cerebrospinal fluid (CSF) of patients with amyotrophic lateral sclerosis (ALS), as well as to show their role in the pathogenesis of ALS experimental models. The level of 25-hydroxycholesterol were higher in untreated ALS patients (n = 30) than in controls without ALS (n = 33) and ALS patients treated with riluzole (n = 9) both in their serum and CSF. The level of 25-hydroxycholesterol in the serum of ALS patients were significantly associated with their disease severity and rate of progression. In the motor neuron-like cell line (NSC34) with the human mutant G93A superoxide dismutase 1 gene (mSOD1-G93A), 25-hydroxycholesterol induced motor neuronal death/ apoptosis via glycogen synthase kinase-3β and liver X receptor pathways; riluzole treatment attenuated these effects. The expressions of enzymes that synthesize 25-hydroxycholesterol were significantly increased in the brains of early symptomatic mSOD1G93A mice. Our data, obtained from patients with ALS, a cellular model of ALS, and an animal model of ALS, suggests that 25-hydroxycholesterol could be actively involved in the pathogenesis of ALS, mostly in the early symptomatic disease stage, by mediating neuronal apoptosis.

## INTRODUCTION

Amyotrophic lateral sclerosis (ALS) is a progressive degenerative disease of the central nervous system (CNS) that lead to the progressive death of the motor neurons [1] and death about four years after disease onset [2]. Although various mechanisms, such as neuroinflammation, excitotoxicity, mitochondrial dysfunction, abnormal protein aggregation, and oxidative stress have been proposed to be associated with the pathogenesis of ALS, no single mechanism has been identified as responsible [3]. Cholesterol, a major constituent of the CNS, is synthesized *de novo* [4]. It is metabolized into diverse types of hydroxycholesterols (OHCs), thereby regulating cholesterol homeostasis [5], cellular apoptosis [6], and signaling [7].

We hypothesized that the synthesis of OHC in the CNS might contribute to the pathogenesis of ALS. The aim of this study was to measure the levels of three major OHCs (24-,25-, and 27-OHCs) in serum (OHC_serum_) and cerebrospinal fluid (OHC_CSF_) of patients with ALS and to assess their role in the pathogenesis of ALS experimental models.

## RESULTS

### Increased levels of OHCs in ALS patients

Patients with ALS who did not receive riluzole treatment (ALS-naive), riluzole-treated patients with ALS (ALS-riluzole), and control groups did not differ significantly in terms of demographic, body mass index, or levels of serum cholesterols (Table 1).

**Table 1:** Basal characteristics of patients

	ALS-naïve	ALS-riluzole	Control	*p*-values		
				ALS-naïve *vs*. control	ALS-naïve *vs*. ALS-riluzole	ALS-riluzole *vs*. control
Number	30	9	33			
Age (yrs)	53.8 ± 12.15 (27.7–79.3)	52.9 ± 6.9 (42.4–66.0)	50.52 ± 16.51 (20.0–74.7)	*n.s*.	*n.s*.	*n.s*.
Male	15 (52%)	4 (44%)	16 (48%)	*n.s*.	*n.s*.	*n.s*.
Total Chol (mg/dL)	182.11 ± 27.78 (112–210)	172.54 ± 30.37 (112–210)	168.45 ± 30.39 (110–225)	*n.s*.	*n.s*.	*n.s*.
BMI	21.75 ± 2.88 (17.09–28.23)	23.14 ± 3.96 (17.24–29.65)	22.71 ± 4.07 (12.83–33.19)	*n.s*.	*n.s*.	*n.s*.
Duration of disease (yrs)	1.00 ± 0.68 (0.25–3.00)	0.93 ± 0.45 (0.36–1.64)		*n.s*.		
ALSFRSr	38.75 ± 6.52 (24–47)	38.89 ± 10.67 (12–48)		*n.s*.		

Abbreviation: ALSFRSr = amyotrophic lateral sclerosis functional rating scale revised, BMI = body mass index, Chol = cholesterol, CSF = cerebrospinal fluid, OHC = hydroxycholesterol.

**Figure 1:**
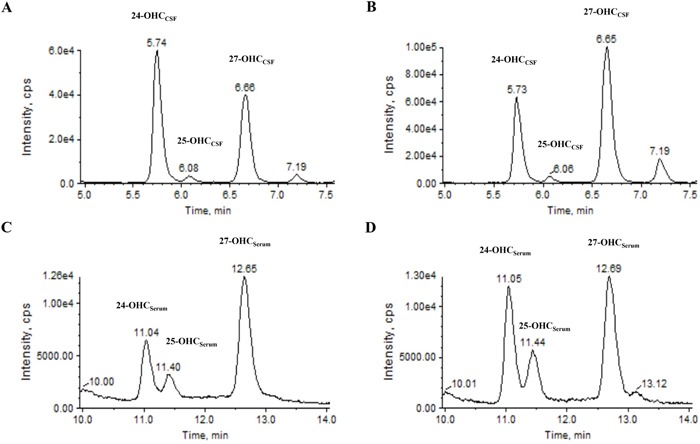
Representative chromatogram of 24-, 25- and 27-OHC in the CSF and serum using ALS patient and control samples For OHC_CSF_ analysis, chromatogram of **A**. normal and **B**. ALS patients were obtained in CSF samples. For OHC_serum_ analysis, chromatogram of **C**. normal and **D**. ALS patient were obtained in serum samples. Abbrevations: CSF = cerebrospinal fluid, min = minutes, OHC = hydroxycholesterol.

**Figure 2:**
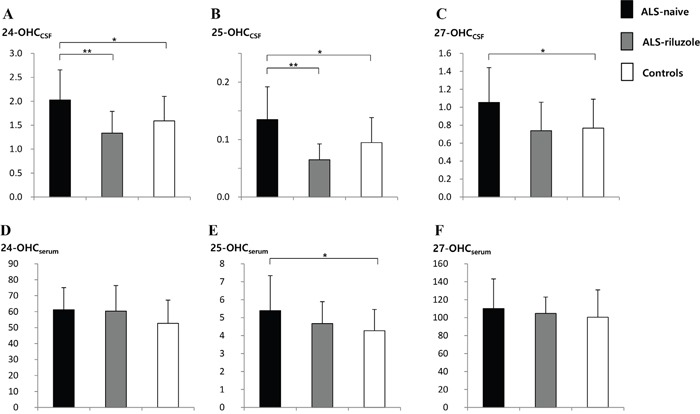
Levels of 24-, 25, and 27-OHC in the CSF and serum of ALS-naïve, ALS-riluzole, and control groups The level of OHCs in the **A-C**. CSF and **D-F**. serum were compared between groups. The levels of (A) 24-OHC_CSF_ and (B) 25-OHC_CSF_ were significantly higher in the ALS-naïve group than both the ALS-riluzole and control groups. The level of (C) 27-OHC_CSF_ and (E) 25-OHC_serum_ were also higher in the ALS-naïve group than in controls. Data for OHCs were expressed in ng/mL. Abbreviations: ALS-naïve = ALS patients without treatment, ALS-riluzole = ALS patients on riluzole treatment, OHC = hydroxycholesterol, OHC_CSF_ = OHC in the cerebrospinal fluid, OHC_serum_ = OHC in the serum. *p < 0.05, **p < 0.01.

The levels of 24-OHC_CSF_ and 25-OHC_CSF_ were significantly higher in the ALS-naïve group (2.03 ± 0.63 ng/mL and 0.14 ± 0.06 ng/mL, respectively) than in the ALS-riluzole group (1.33 ± 0.46 ng/mL, *p =* 0.006 and 0.07 ± 0.03 ng/mL, *p*= 0.001) and controls (1.59 ± 0.05 ng/mL, *p* = 0.018 and 0.09 ± 0.04 ng/mL, *p =* 0.012). The levels of 27-OHC_CSF_ and 25-OHC_serum_ were also higher in ALS-naïve group (1.05 ± 0.39 ng/mL and 5.39 ± 1.94, ng/mL, respectively) than in controls (0.77 ± 0.32 ng/mL, *p* = 0.014 and 4.27 ± 1.18 ng/mL, *p =* 0.017) (Figure 1 and 2).

**Figure 3:**
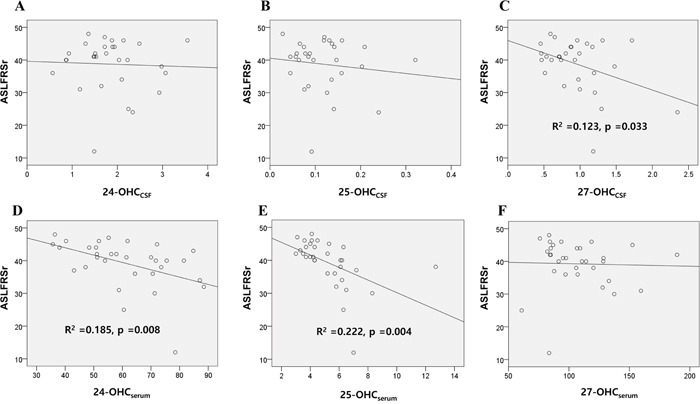
Association of the OHCs with disease severity scores of patients with ALS The association of the 24, 25, and 27-OHC in the **A-C**. CSF and **D-F**. serum of ALS patients with their ALSFRSr were assessed. Univariable linear regression analysis revealed that levels of 27-OHC_CSF_ (C) 24-OHC_serum_ (D) and 25-OHC_serum_ (E) were significantly associated with the ALSFRSr of patients. Data for OHCs were expressed in ng/mL. Abbreviations: ALSFRSr = amyotrophic lateral sclerosis functional rating score revised, OHC_CSF_ = OHC in the cerebrospinal fluid, OHC_serum_ = OHC in the serum.

Disease severity, measured by the revised ALS functional rating scale (ALSFRSr), was significantly correlated with the levels of 27-OHC_CSF_(Figure 3C), 24-OHC_serum_ (Figure 3D), and 25-OHC_serum_ (Figure 3E) in all patients with ALS in the study (n = 37). However, multivariate regression analysis for these three OHCs revealed that only 25-OHC_serum_ level was significantly associated with ALSFRSr score (-0.591; 95% CI -5.385, -0.673; *p* = 0.014).

**Figure 4:**
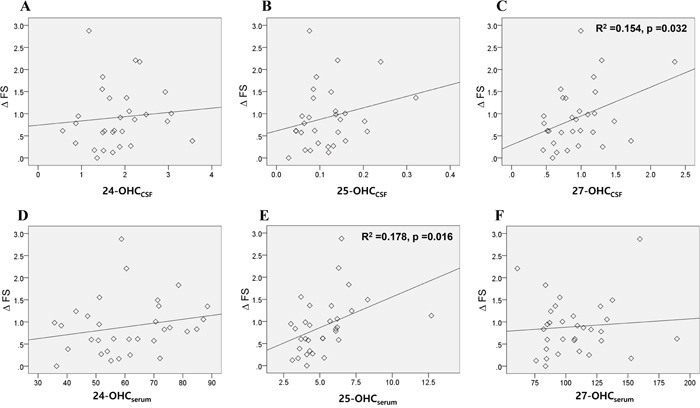
Association of the OHCs with disease progression rate of patients with ALS The association of the 24, 25, and 27-OHC in the **A-C**. CSF and **D-F**. serum of ALS patients with their disease progression rate (ΔFS) were assessed. Univariable linear regression analysis revealed that levels of 27-OHC_CSF_ (C) and 25-OHC_serum_ (E) were significantly associated with the ΔFS of patients. Data for OHCs were expressed in ng/mL. Abbreviations: ΔFS = progression rate in amyotrophic lateral sclerosis functional rating score revised, OHC_CSF_ = OHC in the cerebrospinal fluid, OHC_serum_ = OHC in the serum.

The progression rate (ΔFS), measured by the change of ALSFRSr per months was significantly correlated with the levels of 27-OHC_CSF_ (Figure 4C) and 25-OHC_serum_ (Figure 4E). Multivariate analysis revealed that only the 25-OHC_serum_ level was significantly associated with ΔFS (0.541; 95% CI 0.105, 0.438; *p* = 0.002).

### The 25-OHC induced neuronal apoptosis, activated the GSK-3β pathway in ALS in vitro model, which was attenuated by riluzole

**Figure 5:**
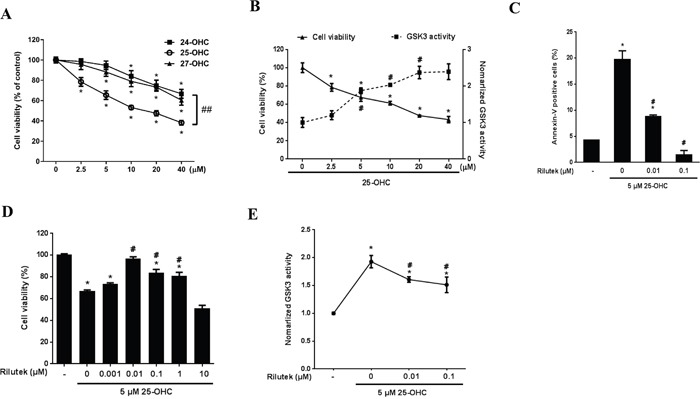
25-OHC induced mSOD1 motor neuronal cell death and activated the GSK-3 pathway **A**. Among the three major OHCS, the 25-OHC induced most severe death of mSOD1-NSC34 cells. **B**. The 25-OHC activated GSK-3 pathways in association with the cellular death and **C**. induced apoptosis. This 25-OHC induced processes, including (C) apoptosis, **D**. cell death, and **E**. activation of the GSK-3 pathways were inhibited by the riluzole treatment. The data are presented as mean (% of the non-treated group) ± SEM and were compared using Tukey's test after a one-way ANOVA (n = 5). *p<0.05 when compared with the non-treated group. #*p*<0.05 when compared with the group that was treated only with 5 μM 25-OHC. ##*p*<0.05 when compared with the 25-OHC treated group. Abbreviations: mSOD1 = mutant superoxide dismutase 1, OHC = hydroxycholesterol.

Among the three OHCs, the 25-OHC induced most severe motor neuronal death in a concentration of the 2.5uM to 40 uM (Figure 5A) in a motor neuronal cell line stably expressing mutant G93A superoxide dismutase 1 gene (mSOD1-NSC34) cells. It also activated the GSK pathway (Figure 5B) and induced cell apoptosis (Figure 5C). Riluzole reduced 25-OHC-induced apoptosis (Figure 5C), neuronal death (Figure 5D), and activation of GSK-3 (Figure 5E).

**Figure 6:**
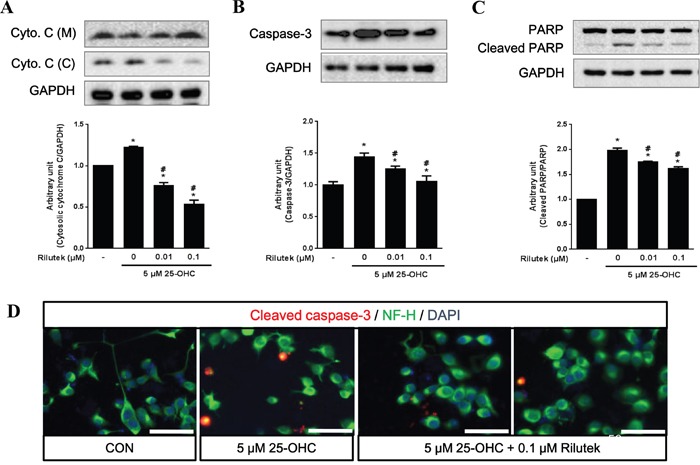
25-OHC-mediated cell death signaling and the effect of riluzole treatment on mSOD1 motor neuronal cells The 25-OHC induced cell death elevated signaling proteins, including **A**. cytosolic cytochrome c, **B**. caspase-3, **C**. and cleaved PARP (A-C). These 25-OHC induced cell death signaling was attenuated by treatment with riluzole. **D**. Immunofluorescence imaging also showed that cleavage of caspase-3 was attenuated by treatment with 0.1uM of riluzole (scale bars = 50 μm). Data were expressed as a ratio of the simultaneously assayed non-treated group's value and were compared using Tukey's test after a one-way ANOVA (n = 5). *p <0.05 when compared with non-treated group. #*p*<0.05 when compared with only 5 μM 25-OHC treated group.

Western blot analysis showed that 25-OHC facilitated release of cytochrome c from injured mitochondria, activated caspase-3, and increased the cleavage of poly (ADP-ribose) polymerase (PARP) in mSOD1-NSC34 cells. However, this activation of cell death signaling cascades by 25-OHC was significantly reduced in a dose-dependent manner after treatment with riluzole (Figure 6A-6C). Moreover, immunofluorescence staining revealed that 25-OHC increased the immunoreactivity of cleaved caspase-3 immunoreactivity and Poly ADP ribose polymerase (PARP), which were reduced by treatment with riluzole (Figure 6D).

**Figure 7:**
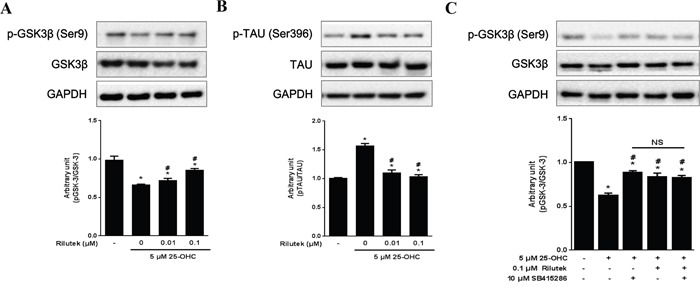
Inhibitory effects of riluzole on the 25-OHC-activated GSK3β pathway in motor neuronal cells Western blot analysis, performed with antibodies against p-GSK3β (Ser9) and its substrate p-TAU (Ser396) revealed that 25-OHC activated A. GSK3β and **B**. subsequent tau pathway. **C**. Both the GSK-3 inhibitor (10 μM SB415286) and riluzole treatments reduced 25-OHC-induced GSK-3β activation. Compounded effects of combined treatment of t GSK-3 inhibitor and riluzole did not differ from the either of these treatments alone (C). Data are expressed as a ratio of the simultaneously assayed non-treated group's value and were compared using Tukey's test after a one-way ANOVA (n=5). *p<0.05 when compared with non-treated group. #*p*<0.05 when compared with 5 μM 25-OHC treated group. NS, not significant.

Administration of 25-OHC have activated of GSK-3β (Ser9) and phosphorylated tau (Ser396), which were prevented by treatment with riluzole (Figures 7A and 7B). Treatment with either GSK-3 inhibitor (10 μM SB415286) or riluzole reduced the GSK-3β activation induced by 25-OHC, although combined treatment with both GSK-3 inhibitor and riluzole did not produce synthetic effects above and beyond the effects of either on its own (Figure 7C).

### 25-OHC induced motor neuronal death via liver X receptor (LXR) signaling

**Figure 8:**
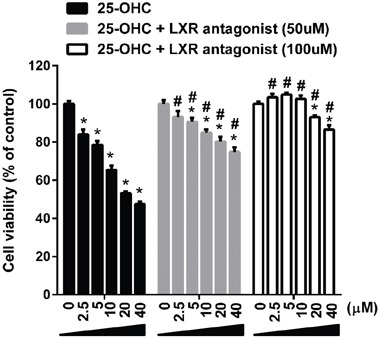
The 25-OHC induced motor neuronal death via LXR signaling Treatment of LXR antagonist (22(S)-OHC; 50uM, 100uM) significantly attenuated the 25-OHC induced death of the mSOD1-NSC34 cells. *p <0.01, when compared with vehicle control. #p <0.01, when compared with the group treated only with the 25-OHC.

To assess whether the 25-OHC mediate motor neuronal death via LXR signaling, a LXR antagonist, 22(s)-OHC [8], were co-treated with the 25-OHC in mSOD1-NSC34 cells. Treatment of 22(s)-OHC significantly attenuated the 25-OHC induced motor neuronal cells in a dose dependent manner (Figure 8).

### Upregulation of the 25-OHC synthesis in ALS mice

**Figure 9:**
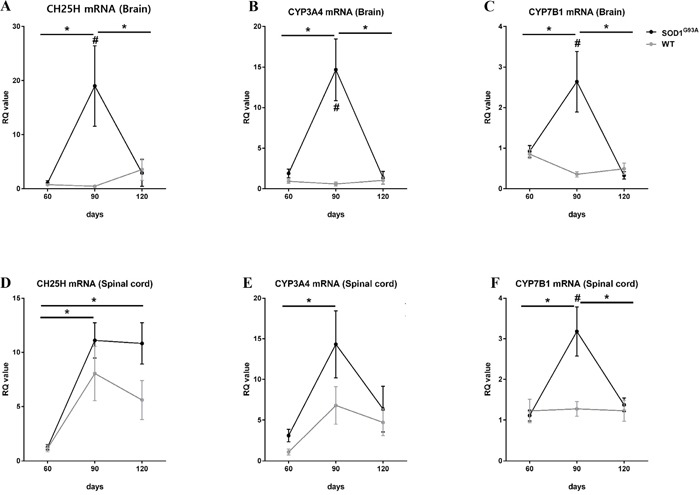
Increased activities of 25-OHC synthesis and metabolism in the early symptomatic stage of SOD1^G93A^ mice The qRT-PCR reactions were performed with mRNA with primers for the 25-OHC synthesizing enzyme (CH25H and CYP3A4) and for the 25-OHC metabolizing enzyme (CYP7B1) using **A-C**. brain and **D-F**. spinal cord at different disease stages (60, 90, and 120 days) of SOD1^G93A^ mice and age-matched WT controls. (A). The expression of CH25H and (B) CYP3A4 mRNA were significantly increased in the brain tissues of early symptomatic stage (90 days) SOD1^G93A^ mice compared to age-matched WT controls and asymptomatic SOD1^G93A^ mice. (C and F). The expression of CYP7B1 was also modestly increased in the brain and spinal cord of SOD1^G93A^ mice in early symptomatic stages. These high expression levels of 25-OHC-associated enzymes were not observed in the late symptomatic stage of SOD1^G93A^ mice. Group means ± SEM are plotted; #*p* <0.05; SOD1^G93A^ mice compared to WT mice on the same day. *p <0.05; SOD1^G93A^ mice compared at different time points. Differences between groups were analyzed with two-tailed Student's t-tests. Abbreviations: CH25H = cholesterol 25-hydroxylase, CYP3A4= Cytochrome P450 3A4, CYP7B1 = 25-hydroxycholesterol 7-alpha-hydroxylase, SOD1 = superoxide dismutase 1, WT = wild type.

In the CNS, 25-OHC is mostly generated by the cholesterol 25-hydroxylase (CH25H) [9] and, to a lesser extent by the cytochrome P450 3A4 (CYP3A4) [10]. Whereas 25-hydroxycholesterol 7-alpha-hydroxylase (CYP7B1) is involved in the metabolism of 25-OHC [11]. To determine a potential mechanism for the observed increases in 25-OHC in ALS, we examined the mRNA expression of CH25H, CYP3A4, and CYP7B1 in the brain and spinal cord of transgenic mice with the human mutant SOD1 gene containing a glycine 93 (Gly93) to alanine (Ala) substation (mSOD1-G93A mice) at different time points (postnatal day of 60, 90, and 120) (Figure 9). In the asymptomatic stage (60 days), the mRNA expression of these enzymes in mSOD1^G93A^ mice did not differ between ALS and WT mice. However, in the early symptomatic stage (90 days), mRNA expression of the 25-OHC synthesizing enzymes, CH25H and CYP3A4, was increased significantly compared to age-matched WT mice and asymptomatic mSOD1-G93A mice; these effects were primarily observed in brain tissue. The 25-OHC metabolizing enzyme, CYP7B1, was also moderately increased in the early symptomatic SOD1G93A mice, both in the brain and the spinal cord. Interestingly, these high levels of mRNA expressions of 25-OHC associated enzymes were only observed in the early symptomatic stage but not in the late symptomatic stage in mSOD1G93A mice.

## DISCUSSION

This study showed that the 25-OHC is involved in the pathogenesis of ALS. The levels of 25-OHC_CSF_ and 25-OHC_serum_ were higher in riluzole-naive ALS patients than in controls and/or riluzole-treated ALS patients. Moreover the level of 25-OHC_serum_ in ALS patients were significantly associated with their disease severity and rate of progressions. The 25-OHC also induced cellular apoptosis and activated the GSK-3β/ LXR pathways in a cellular model of ALS. Lastly the expression of enzymes important for synthesis and metabolism of 25-OHC was increased in an animal model of ALS, in particular, at the early symptomatic stage of the disease.

Our observations are in line with several evidences from the existing literature. Specifically, the expression of the *25-hydroxylase* gene was shown to be increased in autopsied tissues of patients with ALS [12]. The inactivation of liver X receptor beta (LXR*ß*), which promotes cholesterol transport in the CNS, has induced the pathogenesis of ALS pathogenesis in a mouse model [13]. The mutation in the CYP7B1 gene, responsible for 25-OHC metabolism, caused the hereditary degenerative disease involving motor neurons [14]. The metabolism for lipid were dysregulated in both mouse models of ALS and patients with ALS [15, 16]. Finally, 25-OHC can induce mitochondria-dependent cell apoptosis via activation of glycogen synthesis kinase–3*β* (GSK-3*β*) [17], an enzyme considered to be involved in the disease progression of ALS [18].

In a recent study of OHCs in ALS patients, levels of 25-OHC_serum_ tended to be higher in ALS patients than controls, but this difference did not reach statistical significance [19], in contrast to our observations. The reason for this discrepancy may be that our patient inclusion criteria were stringent, such that possible or probable cases of ALS according to the El Escorial criteria were excluded [20]. Moreover, we have controlled the compounding effects of riluzole treatment on OHC level, measured the severity of patients by ALSFRSr, and analyzed the OHC_CSF_, as well as the OHC_serum_, which could have contributed the more robust and readily observable results in our study.

**Figure 10:**
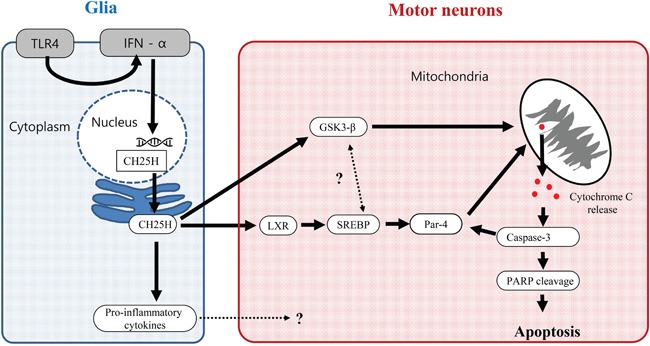
Proposed 25-OHC dependent signaling pathway contributing the motor neuronal death in ALS Abbreviations: CH25H = cholesterol 25-hydroxylase, GSK-3β = glycogen synthesis kinase–3β, IFN = interferon, LXR = liver X receptor, PARP = poly (ADP-ribose) polymerase, PAR-4 = Prostate Apoptosis Response 4, SREBP = sterol regulatory element-binding protein, TLR4 = Toll-like receptor-4.

Among the three major OHCs, the 25-OHC induced most severe motor neuronal death in vitro model of ALS compared to the 24- and 27-OHC (Figure 5A). The 25-OHC is produced in the macrophage and/or glia by the enzyme CH25H [21, 22], in response to Toll-like receptor-4 (TLR-4) signaling [23] Moreover patients with ALS had increased expression of the TLR-4 in their glia of spinal cord. [24]. We speculate that this 25-OHC could mediate the motor neuronal death of ALS, via GSK3-ß and LXR pathway, in response to the upregulated the inflammatory signals of the glia (Figure 10).

The OHC engaged in diverse biological activities, including cell signaling, cellular cholesterol synthesis, and cytotoxicity [25, 26]. Among these metabolites, the level of 24-OHC has been shown to increase in patients with Alzheimer's disease and is associated with disease severity [27]. The metabolite 27-OHC is proposed to reflect the effect of the peripheral hypercholesterolemia to the CNS via its net flux from peripheral circulation to the CNS [28]. Recently, 25-OHC has drawn attention for its role as both an anti-inflammatory and pro-inflammatory mediator [11]. In addition to these important roles of OHCs, we speculated that 25-OHC might play a pathogenic role in ALS by mediating GSK-3ß activation, LXR signaling, and neuronal apoptosis.

Although disease severity in patients with ALS patients was correlated with levels of 25-OHC_serum_, it was not correlated with levels of 25-OHC_CSF_. This may explained by our finding that the expression of 25-OHC synthesizing enzyme was only transiently increased in the CNS of ALS mice during the early symptomatic stage of the disease. It may be that this temporarily high expression of 25-OHC-synthesizing enzymes may have interfered with the production of 25-OHC in the CNS of patients who are in the advanced stages of ALS.

The precise cause of decreased 25-OHC_CSF_ in ALS-riluzole patients remains unclear. However, in light of a previous study showing that glutamate excitotoxicity increased both cholesterol and cholesterol oxidation products in the brain [14], these results may be attributable to anti-glutamatergic effects of riluzole [29].

Our study has several limitations. First, the number of patients were relatively small. Second, the 25-OHC may not be solely responsible for the whole pathogenesis of ALS. Because the ALS-riluzole group, who had relatively low levels of 25-OHC_CSF_, continued to experience the disease progression [30]. Moreover, the 25-OHC might also mediate diverse disease conditions, including viral infection [31], X-linked adrenoleukodystrophy [8], multiple sclerosis [32], and neuromyelitis optica [33], other than the ALS [23]. Therefore, it seems that the 25-OHC might be one of the diverse inflammatory mediator rather than a specific marker for ALS. Second, we have used the serum and CSF from patients as controls, rather than those from healthy volunteers. This is for the ethical issues of performing the lumbar punctures on the healthy subject. However, as we have excluded both the degenerative or inflammatory diseases of the CNS from the controls, we speculate that this issue would not significantly affect the result of our study. Lastly, the level of OHCs in the CSF and serum of ALS animal could also have been another interesting point, which were not performed in our study because the concentrations of the OHC were expected to be extremely low in mice.

Our data showed that the 25-OHC could be an important mediator of the ALS pathogenesis involving in the GSK3-ß activation and neuronal apoptosis, which could eventually trigger the onset or accelerating the progression of ALS symptoms. Our results may reveal a novel ALS treatment target and provide insight into the involvement in of CNS cholesterol metabolism in ALS pathogenesis.

## MATERIALS AND METHODS

### Patients

Patients who conformed to the diagnosis of definite or probable ALS according to the revised El Escorial criteria [20] were recruited from the ALS Clinic at the Seoul National University Hospital between December 2009 and May 2012. The following patients were excluded: those diagnosed more than 3 years before study commencement, those aged < 20 years or >80 years, those with respiratory support (tracheostomy, non-invasive ventilation, or oxygen inhalation), those fed via levin tube or gastrostomy tube (percutaneous endoscopic gastrostomy, PEG), those with presence of an acute systemic illness (per se, aspiration pneumonia), previous enrollment in the clinical drug or stem cell trial for ALS, or absence of written consent. ALS patients were also dichotomized according to the use of riluzole, as ALS-naive group and ALS-riluzole group [30].

The control group was comprised of patients who visited the Neurology Clinic at the Seoul National University Hospital. Patients were excluded from the control group if they had been diagnosed with a either neurodegenerative [20] or neuroinflammatory disease [34] of the central nervous system, any clinical or electrophysiological evidence of motor neuron disease [20], or if they had used an oral or intravenous steroid that might have affected cholesterol metabolism.

### Clinical parameter and sampling protocols

Age, gender, level of serum total cholesterol, body weight, and height were assessed in patients with ALS and non-ALS controls. The ALSFRSr [35] and duration of disease were also assessed in patients with ALS. The progression rate (ΔFS) was calculated with minor modification of the previous study as follows; Δ FS = (48 - ALSFRSr at “time of sampling”)/duration from onset to diagnosis (month) [36]. All samples of CSF and serum were immediately centrifuged and stored at – 80°C after collection, according to standard protocols [37].

### Measurement for the CSF and serum level of 24-, 25-, and 27-hydroxycholesterols

Liquid chromatography-tandem mass spectrometry (LC–MS/MS) analyses were performed at Korea Institute of Science and Technology (KIST), according to our recent methods [33]. Briefly, an API 4000 triple-quadrupole mass spectrometer (AB Sciex, Toronto, Canada) with an electrospray ionization source, in the positive ionization mode was used. The electrospray source was coupled online with a Shimadzu ultra-fast LC system (Shimadzu Corporation, Kyoto, Japan).

### Standard protocol approval, registration, and patient consent

This study was approved by the Seoul National University Hospital Institutional Review Board (IRB number: H-0904-057-279). All patients provided written informed consent prior to participation. This study was approved by the IACUC (Institutional Animal Care and Use Committee) of Hanyang University.

### Cell culture, treatment, and the GSK-3 activity

The NSC-34 mouse motor neuron cell line [38] was purchased from CELLutions (CELLutions Biosystems Inc., Ontario, Canada), and the cells were grown in the high glucose formulation of DMEM (D5796; Sigma, USA) supplemented with 10% fetal bovine serum (FBS) and 1% PenStrp. To induce differentiation of the NSC-34 cells (dNSC-34), they were grown to confluence, and the medium was replaced with a fresh medium consisting of a 1:1 mixture of DMEM with Ham's F12 containing 1% FBS, 1% PenStrp, and 1% modified Eagle's medium non-essential amino acids, and were maintained for 3 days until use [39]. All media were purchased from GIBCO (GIBCO, USA). We stably overexpressed wild and mutant G93A superoxide dismutase 1 (SO*D*1, NM_000454) in NSC-34 cell lines that were selected using 100 μg/mL geneticin [40]. Exogenous 22(S)-, 24-,25-, and 27-OHC (Sigma, USA) was dissolved in ethanol [41]. The final concentration of ethanol in media did not exceed 0.1%. Riluzole was provided by Sanofi-aventis (Korea). Cells were pre-treated with several concentrations of 25-OHC (0, 2.5, 5, 10, 20 μM, and 40 μM) alone for 24 h, then treated with riluzole (0, 0.01, 0.1, 1, 10 μM, and 100 μM) for 24 h, and then washed carefully several times with phosphate-buffered saline (PBS). Cell viability was assessed by the MTT assay [42]. GSK-3 activity was evaluated using GSK-3 substrate phosphoglycogen synthase peptide-2 (Upstate, USA) as described previously [43].

### Immunoblot analysis

After chemical treatment, cells were lysed with RIPA buffer supplemented with phosphatase inhibitor. Cell lysates were subjected to immunoblotting with antibodies; p-Akt (Ser473) (1:500, Cell Signaling Technology, Danvers, MA, USA), Akt (1:1000, Cell Signaling), p-GSK-3β (Ser9) and GSK-3β (1:1000, Santa Cruz Biotechnology, Dallas, Texas, USA), cytochrome c (1:500, Santa Cruz Biotech), PARP (1:1000, Santa Cruz Biotech), caspase-3 (1:1000, Cell Signaling), p-Tau (Ser396) and Tau (1:1000, Invitrogen), and GAPDH (1:1000, Santa Cruz Biotechnology). To evaluate cytosolic cytochrome c levels, cell pellets were fractionized using the Qproteome cell compartment kit (Qiagen, Germantown, MD, USA). The reactive bands were detected by ECL (Amersham Pharmacia Biotech, Piscataway, NJ, USA) and quantified with an image analyzer (Bio-Rad, Quantity One-4, 2, 0). The same membranes were probed for GAPDH as an internal control [44].

### Immunocytochemistry

Briefly, the cells were washed with PBS, fixed in 4% formaldehyde, and permeabilized with 0.1% Triton X-100 for 5 min. Primary antibodies used were as follows: NF-H (neurofilament heavy chain, 1:2000, Abcam, Cambridge, MA, USA) and cleaved caspase-3 (1:200, Cell Signaling). For detection, cells were labeled with fluorescent secondary AlexaFluor 488-labelled anti-chicken and TRITC-conjugated anti-rabbit antibodies (1:500, Molecular Probes, Invitrogen, USA) for visualization. Slides were cover-slipped with a drop of DAPI medium (Sigma, Saint Louis, MO, USA). Cells without the primary antibody served as negative controls.

### Evaluation of apoptotic cells

To evaluate apoptosis, cells were stained by annexin V-FITC apoptosis detection kit (Sigma) following the manufacturer's instructions. Cells were washed and briefly trypsinized, and then washed twice with cold PBS. Cells were pelleted by centrifugation, resuspended in 1X binding buffer, and incubated with a staining solution (annexin V-FITC and PI) for 15 min. in the dark at 4°C. The cells were then resuspended in 1X binding buffer. Samples were kept on ice during the entire procedure and analyzed immediately by flow cytometry. Ten thousand cells from each sample were scanned and analyzed by FACS Canto (Becton Dickinson) using the standard configuration and parameters. Data acquisition and analysis was performed using the FACS DIVA software (BD Biosciences, San Jose, California, USA). Necrosis and apoptosis were determined by PI (FL2) and annexin V-FITC (FL1) fluorescence, respectively.

### Transgenic SOD1G93A mice

B6SJL-Tg (SOD1-G93A) 1Gur/J mice were obtained from the Jackson Laboratory (Bar Harbor, ME, USA). The mice were housed under a 12-h light/dark cycle and bred following the supplier's protocol. The presence of the human G93A transgene was confirmed by polymerase chain reaction (PCR) and evaluated for transgene copy numbers [45]. In this strain, the first symptoms and end-stage symptoms of ALS appear at approximately 77 and 136 days of age [46]. Thus, we divided the SOD1^G93A^ mice into three groups that were evaluated at the following time points: 60 (asymptomatic), 90 (early symptomatic), and 120 days of age (late symptomatic) [47]. Wild-type (WT) mice (B6SJLF1/J) obtained from the Jackson Laboratory were used as controls. All animal experiments were approved by the Institutional Animal Care and Use Committee of Hanyang University.

### Real-time polymerase chain reaction (RT-PCR)

We quantified mRNA expression of CH25H and CYP3A4, which synthesize 25-OHC, and the CYP7B1, which metabolizes 25-OHC, using the mSOD1G93A mice (n = 30) and age-matched WT mice (n = 30). Mice were randomly assigned into three groups that were evaluated at each time points: n = 10. RNA was isolated from the lumbar spinal cord and cortical brain regions of mice and extracted using RNeasy Mini Prep (Qiagen, Germany). The quantity and integrity of RNA was evaluated by measuring the absorbance at 260 nm via Nanodrop (Thermo Scientific, ND-2000). The cDNA was synthesized from 2.5 μg of RNA using Vilo cDNA kit (Invitrogen, USA) per the manufacturer's protocol. Primers for quantitative polymerase chain reaction (PCR) were purchased from Qiagen. We analyzed CYP3A4 (Qiagen, Germany, PPM03943B), CYP7B1 (Qiagen, Germany, PPM03979F), CH25H (Qiagen, Germany, PPM27829A), and GAPDH (Qiagen, Germany, PPM02946E). First-strand cDNA was amplified using Power SYBR Green PCR master mix (Applied Biosystems, USA) with primers. Real-time RT-PCR was conducted with Applied Biosystems StepOnePlus^TM^ (Carlsbad, USA) at 95°C for 10 min, followed by 40 cycles of 15 sec at 95°C and 1 min at 60°C. A melting curve was performed to check the specificity of amplification. The relative quantity (RQ) was calculated by 2-ΔΔCt, taking GAPDH as the interval standard control. Each sample was analyzed in triplicate.

### Statistical analyses

Clinical data are presented as means ± standard deviation. Experimental data are presented as means ± SEM of five or more independent experiments. Differences between groups were analyzed by Student's t-test or one-way ANOVA followed by Tukey's post hoc comparisons. Two-tailed *p*-values < 0.05 were considered statistically significant. Linear regression analyses were used to test for associations between values. All statistical analyses were performed using the SPSS 17.0 software package for Windows (SPSS, Seoul, Korea).

## Abbreviations

ALS: amyotrophic lateral sclerosis
ALSFRSr: amyotrophic lateral sclerosis functional rating scale revised
CH25H: cholesterol 25-hydroxylase
CNS: central nervous system
CSF: cerebrospinal fluid
CYP3A4: cytochrome P450 3A4
CYP7B1: 25-hydroxycholesterol 7-alpha-hydroxylase
GSK: glycogen synthase kinase
HSP: hereditary spastic paraplegia
OHC: hydroxycholesterol
PARP: poly (ADP-ribose) polymerase
PBS: phosphate-buffered saline
SOD1: superoxide dismutase 1
